# The human milk microbiome is minimally associated with breastfeeding practices

**DOI:** 10.1038/s41598-025-03907-7

**Published:** 2025-06-02

**Authors:** Ruomei Xu, Mark P. Nicol, Ali S. Cheema, Jacki L. McEachran, Ashleigh H. Warden, Sharon L. Perrella, Zoya Gridneva, Donna T. Geddes, Lisa F. Stinson

**Affiliations:** 1 School of Molecular Sciences, The University of Western Australia, 35 Stirling Hwy, Crawley, WA 6009 Australia; 2 UWA Centre for Human Lactation Research and Translation, Crawley, WA 6009 Australia; 3 ABREAST Network, Perth, WA 6000 Australia; 4 School of Biomedical Sciences, Marshall Centre, The University of Western Australia, Crawley, WA 6009 Australia; 5 The Kids Research Institute Australia, Nedlands, WA 6009 Australia

**Keywords:** Lactation, Human milk, Breastfeeding, Microbiome, Infant feeding, Breast pumps, Bacteria, Microbial communities

## Abstract

The human milk microbiome is dominated by typical oral and skin bacteria, suggesting that bacterial communities from the infant mouth and maternal skin contribute to the development of the human milk microbiome. It is postulated that breastfeeding characteristics, such as breastfeeding frequency and duration, could lead to different levels of exposure to oral and skin bacteria, and subsequently, altered bacterial profiles in human milk. To investigate the associations between breastfeeding characteristics and the human milk microbiome, this study analysed milk samples collected from 56 participants at 3 months postpartum by full-length 16 S rRNA gene sequencing. Breastfeeding characteristics (breastfeeding frequency, total 24 h breastfeeding duration, and 24 h milk removal volume) were recorded by the participants using the 24 h test weighing protocol. The milk microbiome was largely robust to breastfeeding practices, with only one association detected between breastfeeding characteristics and the milk microbiome. Duration of breastfeeding from the sampled breast (per 24 h) was weakly positively associated with the relative abundance of *Streptococcus salivarius* (*P* = 0.035). No associations with alpha nor beta diversity were detected. In conclusion, variations in breastfeeding characteristics do not have a major impact on the composition or diversity of the human milk microbiome.

**Supplementary Information:**

The online version contains supplementary material available at 10.1038/s41598-025-03907-7.

## Introduction

Human milk contains many microorganisms including *Staphylococcus*, *Streptococcus*, *Acinetobacter*, and *Cutibacterium* species^1,2^. The origins of the human milk microbiome are thought to include the bacterial communities from the infant oral cavity and maternal skin, since a large number of typical oral and skin bacteria are detected in human milk^2^. This hypothesis was further supported by a previous study which found that mothers who fed their infants with expressed milk had a less rich human milk microbiome compared to mothers who fed the infants directly at the breast^3^. Another study suggested that breastfeeding frequency and total 24 h breastfeeding duration could be associated with specific bacterial taxa including *Bifidobacterium*^4^. According to recommendations from the Centers for Disease Control and Prevention, the majority of fully breastfed infants need to be fed every 2–4 h, resulting in 8–12 breastfeeding sessions per day in the first few months^5^. However, even within fully breastfeeding dyads, there is a wide range of normal breastfeeding characteristics, including time spent breastfeeding, volume of milk produced, and number of breastfeeds. There is little evidence regarding the impact of such characteristics on the human milk microbiome. Therefore, this study aimed to investigate how breastfeeding frequency, total 24 h breastfeeding duration, and 24 h milk production were associated with human milk bacterial profiles.

## Results

The characteristics of the mothers included in the analysis are presented in Table 1. The majority of mothers fully breastfed their infants at the time of sample collection (96.4%).

**Table 1:** Participant characteristics and milk production parameters (*n* = 56).

Characteristic	Mean ± SD or *n* (%)
Maternal	
Age at delivery (years)	32.6 ± 4.8
Ethnicity	
Caucasian	50 (89.3)
Other	6 (10.7)
Pre-pregnancy BMI category*	
Underweight	2 (4.7)
Normal weight	27 (62.8)
Overweight	8 (18.6)
Obese	6 (14.0)
Parity	
1	12 (21.4)
2	28 (50.0)
> 2	16 (28.6)
Delivery mode	
Vaginal	40 (71.4)
Caesarean section	16 (28.6)
Intrapartum antibiotic prophylaxis**	
Yes	23 (41.8)
No	32 (58.2)
Breast pump use (in previous month)	
Yes	14 (25.0)
No	42 (75.0)
Infant	
Birth gestation (weeks)	39.3 ± 1.1
Birth weight (g)	3523.0 ± 379.3
Weight at 3 months postpartum (g)	6142.0 ± 749.3
Sex	
Female	29 (51.8)
Male	27 (48.2)
Feeding mode (in previous month)	
Full breastfeeding	54 (96.4)
Mixed	2 (3.6)
24 h milk profile	
Total breastfeeding duration, sampled breast (min)	83.8 ± 43.0
Breastfeeding frequency, sampled breast	5.3 ± 1.7
24 h milk removal volume, sampled breast (g)	415.1 ± 152.6
Breast pump use (during milk profile analysis)	
Yes	3 (5.4)
No	53 (94.6)
Formula use (during milk profile analysis)	
Yes	2 (3.6)
No	54 (96.4)

*13 missing records.

**1 missing record.

**Fig. 1:**
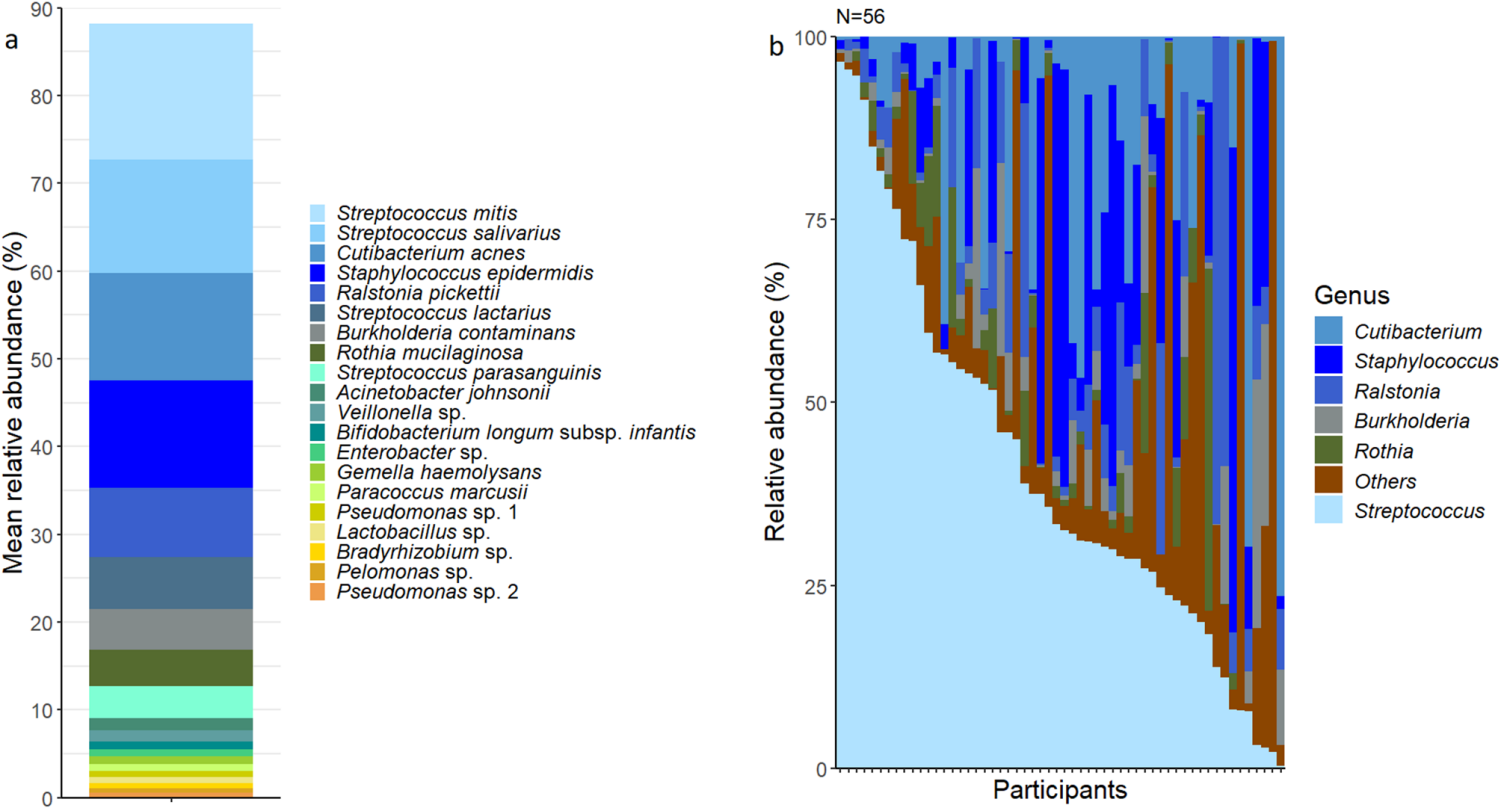
(a) Composition of the human milk microbiota at 3 months postpartum. Operational taxonomic units (OTUs) with a mean relative abundance of ≥ 0.5% are displayed; (b) Genus-level inter-individual variation in milk bacterial profiles of the 56 participants. Genera that comprised < 2% are grouped together as “other”.

In these human milk samples (collected at 3 months postpartum), 573 bacterial genera and 30,290 operational taxonomic units (OTUs) were detected. A total of 20 bacterial taxa with relative abundance ≥ 0.5% were identified, representing 88% of the bacterial sequences recovered from the samples. The human milk microbiota was dominated by typical skin and oral taxa including *Streptococcus mitis* (mean relative abundance 15.6% ± 20.6%), *Streptococcus salivarius* (12.9% ± 19.3%), and *Cutibacterium acnes* (12.3% ± 17.6%) (Fig. 1a) with a high level of inter-individual variation (Fig. 1b).

Given that the breastfeeding characteristics analysed here (breastfeeding frequency, breastfeeding duration, and volume of milk removed) are likely interrelated, we tested for correlations between these factors. Breastfeeding frequency was positively correlated with 24 h breastfeeding duration (*r* = 0.299, *P* = 0.003) and 24 h milk removal (*r* = 0.310, *P* = 0.002). However, there was no correlation between 24 h breastfeeding duration and 24 h milk removal (*r* = 0.117, *P* = 0.206). Since samples were only collected from one breast, we restricted our analysis of breastfeeding characteristics to the sampled breast only. However, it should be noted that breastfeeding characteristics did not differ between the sampled and non-sampled breast (Student’s t-test, total 24 h breastfeeding duration: *P* = 0.175; breastfeeding frequency: *P* = 0.297; 24 h milk removal volume: *P* = 0.265).

**Fig. 2:**
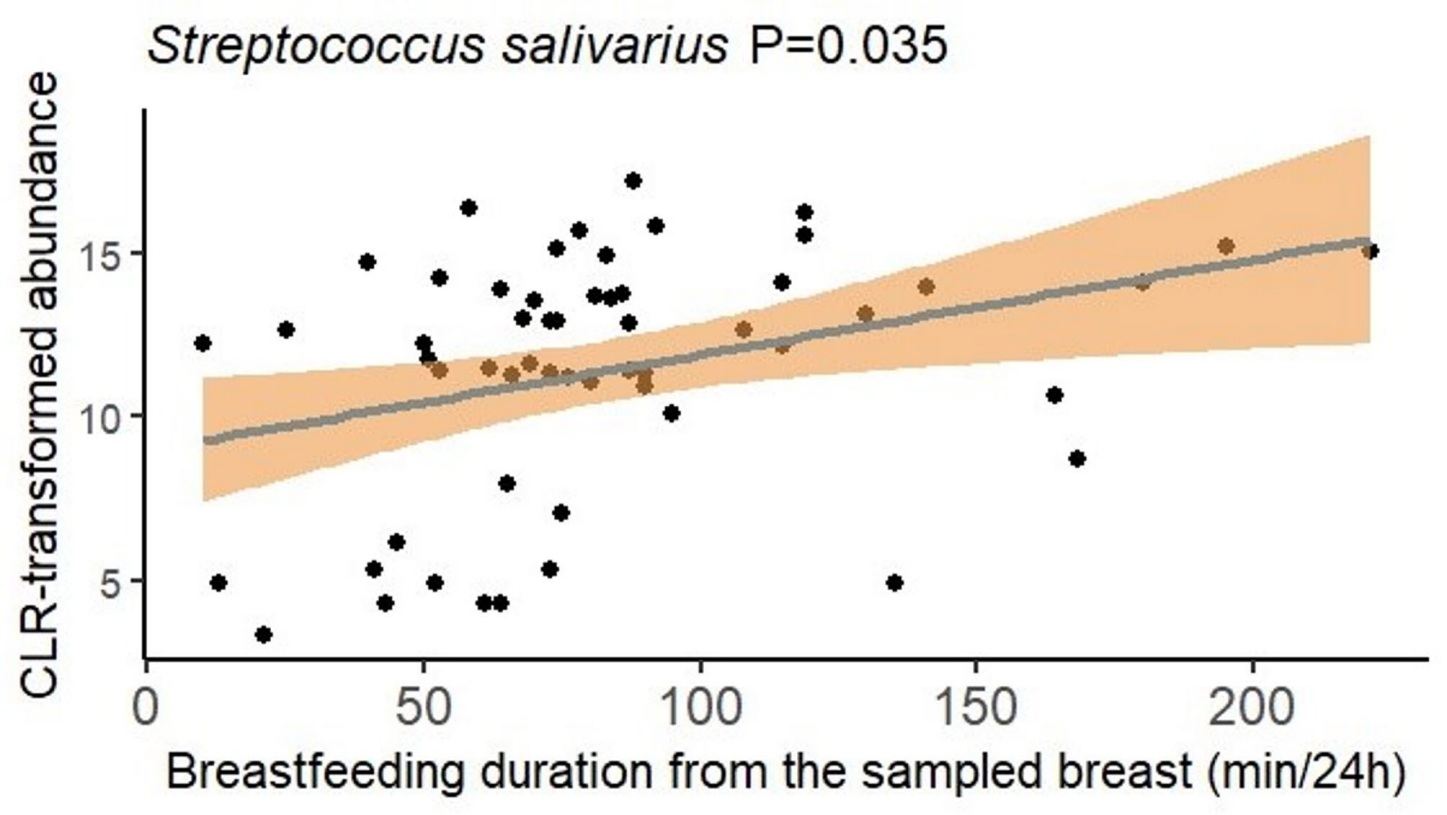
Duration of breastfeeding from the sampled breast was positively associated with the relative abundance of *S. salivarius*. Lines represent linear models fitted to the data with shaded areas representing 95% confidence intervals.

Overall, only a single breastfeeding characteristic was associated with a small, though potentially biologically relevant, alteration to the human milk microbiome (Supplementary Table 1). Twenty-four-hour breastfeeding duration from the sampled breast was weakly positively associated with the relative abundance of *S. salivarius* (coefficient = 0.026, *P* = 0.035; Fig. 2). No other associations were detected for the other 19 OTUs analysed here, nor for Shannon diversity or richness of the human milk microbiome (Supplementary Fig. 1). Similarly, breastfeeding characteristics were not associated with the Bray-Curtis distance between the human milk samples.

## Discussion

This pilot study provides new data demonstrating that the human milk microbiome is largely unaffected by breastfeeding practices. We found that at 3 months postpartum, the total duration of breastfeeding per 24 h was positively associated with the relative abundance of *S. salivarius*. However, it is important to note the small effect size of this association (coefficient = 0.026). None of the breastfeeding characteristics analysed here were associated with Shannon diversity, richness, or the beta diversity of the human milk microbiome. Thus, our findings indicate that the human milk microbiome is largely robust to variations in breastfeeding characteristics, with no structural changes, despite a single minor variation in composition.

In our study, a longer duration of breastfeeding was associated with a greater relative abundance of *S. salivarius* in human milk. As *S. salivarius* has been demonstrated to be a part of the healthy infant oral microbiome^2,6,7^, and milk from the infant oral cavity may flow back into the mammary gland during breastfeeding^8^, this finding is biologically plausible. It is postulated that there is bidirectional exchange of bacteria between the infant oral cavity and the lactating mammary gland^9,10^. This theory is supported by data from a previous study of Guatemalan mothers, showing a higher level of typical oral bacteria, including *Streptococcus* species, in mothers who exclusively breastfed compared to those who mix-fed^11^. However, other strain-level evidence demonstrates that typical oral species, including *Streptococcus* species, are present in pre-colostrum prior to contact with the infant oral cavity^12^. Thus, these taxa are likely true colonisers of the mammary gland, with their abundance potentially bolstered by interaction with the infant oral cavity. Given the paired t-test results showing no difference in the breastfeeding characteristics between left and right breasts in the individual participants, these findings could be representative of the other breast which we did not take samples from. Breastfeeding duration and breastfeeding frequency were positively correlated in this study. It is therefore perhaps surprising that breastfeeding frequency was not associated with the relative abundance of *S. salivarius*. This observation may suggest that it is the amount of time that the mammary gland is exposed to the infant oral cavity, as opposed to the number of bouts of exposure, that matters. This theory makes sense from a microbial ecology standpoint. Future studies with larger sample sizes are needed to investigate further the associations between exposure to the infant oral microbiome and the abundance of oral bacteria in the milk microbiome.

Our study did not find any associations between breastfeeding characteristics and the diversity of the human milk microbiome. In a recent study on 46 mother-infant pairs living in the United States (US), breastfeeding frequency was found to be negatively associated with milk microbiome richness^4^. Given that longer time spent breastfeeding and higher breastfeeding frequency would lead to the mammary gland having more exposure to the bacterial communities from maternal skin and infant mouth, which may lead to a more diverse microbiome in human milk, these findings were unexpected. It is important to note that, although infants feed at night, the US study only observed breastfeeding from 7 am to 7 pm. In addition, the recording of breastfeeding ‘bouts’ (defined as breastfeeding sessions separated by > 30 seconds) could artificially increase the breastfeeding frequency analysed. Therefore, the findings of this study need to be interpreted with caution. Another factor to consider in interpreting both the results of the present study and the US study is the use of 16S rRNA gene sequencing. Since this methodology provides relative abundance rather than absolute quantification of microorganisms, it is possible that repeated exposure to skin and oral bacteria increases the absolute abundance of certain bacteria thereby reducing the likelihood of identifying associations with lower-abundance bacteria^13^. Therefore, quantitative methods such as qPCR may be useful to further investigate associations between breastfeeding practices and the structure of the human milk bacterial communities.

This study has several strengths. Firstly, the majority of the participants fully breastfed their infants, which reduced the possibility of formula use altering the human milk microbiome as a confounder. We also prospectively collected real-time data on breastfeeding characteristics to avoid recall and observer bias. Further, the use of full-length 16 S rRNA gene sequencing allowed us to investigate the human milk microbiota at the species level. Despite these strengths, this study also has limitations. Despite best practice to limit reagent-based contamination^14^, we identified some taxa in our milk profiles that were likely to be contaminants, namely *Burkholderia*, *Ralstonia*, and *Bradyrhizobium*. Reagent-based contamination is ubiquitous in microbiome data and is especially problematic for low-biomass samples such as human milk. Secondly, the sample size of our analysis was relatively small and contained mainly Caucasian and multiparous women. Given that breastfeeding characteristics vary geographically^4,15^, the findings of this study may not be representative of other populations, especially in low- and middle-income countries. Thirdly, data on breastfeeding characteristics were only available at 3 months postpartum, so the findings of this study may not be generalised beyond the first few months of lactation. Finally, volume of milk removed from the sampled breast was used as a proxy for 24 h milk production of the breast, since milk production data were calculated from both breasts^16^. Therefore, the associations with milk removal from the sampled breast might not accurately reflect the associations between milk production and the human milk microbiome at the individual breast level. Additionally, there are other breastfeeding practices that were not analysed here that would be valuable to investigate, including hand/pump expression, pumping frequency, breast pump cleaning practices, and allomaternal feeding. Future longitudinal studies with larger sample sizes, human milk samples and milk production data collected for both breasts, as well as the inclusion of more feeding and milk removal practices are needed for in-depth investigations into the associations between breastfeeding characteristics and the human milk microbiome.

In conclusion, this study identified a single minor change in the composition of the human milk microbiome associated with breastfeeding characteristics, suggesting that variance in breastfeeding practices largely does not alter the milk microbiome.

## Methods

### Study design and sample collection

This study included mothers from the Breastfeeding Longitudinal Observational Study of Mothers and kids (BLOSOM) birth cohort. Cohort recruitment, inclusion and exclusion criteria have previously been reported^17^. Briefly, healthy mother-infant dyads were included, with all mothers in this cohort fully (*n* = 54) or predominantly (*n* = 2) breastfeeding at the 3-month sample time point. Written informed consent was obtained from all participants, and the study was approved by the Human Research Ethics Committee of The University of Western Australia (RA/4/20/4023). All methods were performed in accordance with the relevant guidelines and regulations.

As described previously^2^, the participants chose one breast to donate milk samples over the study period. The human milk samples were self-collected aseptically by the participants and stored in their home freezer for up to 18 h, before being transported to the laboratory and stored at -80⁰C until further analysis.

### Breastfeeding characteristic data collection

Breastfeeding characteristics were recorded at 3 months postpartum by the participants using the 24 h test weighing protocol^18^. Given that milk was only sampled from one breast, our analysis focused on breastfeeding characteristic data from that breast only. However, milk production is necessarily calculated using data from both breasts, therefore we used total 24 h milk removal (g) from the sampled breast as a proxy for 24 h milk production of the breast. Twenty-four-hour milk removal from the sampled breast was calculated by summing the differences in weight of the infant before and after every breastfeed and differences in weights of milk collection bottles before and after every pumping session over 24 h. Each feed from a single breast was counted as one breastfeed. The total 24 h breastfeeding duration (minutes) was summed from the duration of all recorded breastfeeds from the sampled breast. Mothers with complete human milk microbiome and breastfeeding characteristic data available at 3 months postpartum were included in the analysis (*n* = 56).

### Microbiome analysis

The 56 samples used in the microbiome analysis were a subset of those from a broader cohort (539 human milk samples collected at 9 time points from 84 mothers). All these samples were analysed in 96 well-format batches, consisting of 94 samples, one negative extraction control, and one negative amplification control. In total, seven plates (batches) were used. To avoid batch effects, samples were randomised across the plates. Total DNA was extracted from 1 mL aliquots of milk using the QIAGEN MagAttract Microbial DNA Isolation Kit on the Kingfisher Flex platform. The full-length 16 S rRNA gene was amplified in 30 µL reactions consisting of: 1X AccuStart II PCR ToughMix, 0.3 µM each of the PacBio barcoded forward (27 F) and reverse (1492R) primers, 0.75 µL each of dsDNase and DTT from the ArcticZymes PCR decontamination kit, 3 µL nuclease-free water, and 6 µL of template. The PCR cycling conditions consisted of an initial heating stage of 94 °C for 3 min, followed by 38 cycles of 94 °C for 30 s, 52 °C for 30 s, and 72 °C for 2 min 30 s and a final extension stage of 72 °C for 5 min. Amplicons were multiplexed in equimolar concentrations, and Macherey Nagel NucleoMag NGS beads were used to concentrate and purify the multiplexed pools. Sequencing was performed on the PacBio Sequel II platform at The Australian Genome Research Foundation (AGRF).

Sequence data processing was performed using mothur v.1.48.0^19^. Sequence quality filtering was performed based on length (1336–1742 bp), alignment (position 1044–43116), and homopolymers (≤ 9). The SILVA reference alignment v132 was used to align the sequences^20^. VSEARCH was used to exclude the chimeric sequences after alignment^21^. OTUs were generated using the cluster split method with a cutoff distance of 0.03. Subsampling was performed at 1999 reads, resulting in an average sequence coverage of 97%. Our subsampling method resulted in the exclusion of 3 low-yield samples. The remaining samples had an average sequence read count of 11,878 ± 7,754 and a median of 10,538. Alpha diversity was assessed by richness and Shannon diversity, and beta diversity was assessed using Bray-Curtis distance. Compositional analysis was performed at the OTU level, with OTUs with a mean relative abundance of ≥ 0.5% included in the analysis (*n* = 20). OTU abundance data was centred log ratio (CLR) transformed. Initial genus-level taxonomic assignment was mapped using the SILVA taxonomy database (v132), with species-level assignment manually performed using BLAST^22^. To assign species-level taxonomy with BLAST, the top hit for sequences generated using whole genome or long-amplicon 16 S rRNA gene sequencing was selected, resulting in sequence identity score of ≥ 98.25% for all assignments. Genus-level identity is provided in cases of OTUs having an equally good match for more than one bacterial species, or in cases where all good matches (> 90% identity score) were at the genus-level or above. BLAST-derived taxonomy including percent ID and coverage data are provided in Supplementary Table 2.

Reads recovered from negative extraction and amplification controls are listed in Supplementary Table 3.

### Statistical analysis

Kendall’s Tau correlations were used to assess correlations between breastfeeding characteristics. Paired t-tests were used to analyse the difference in the breastfeeding characteristics between left and right breasts in the individual participants.

In this small investigative study, we modelled associations between the human milk microbiome and breastfeeding characteristics using linear models individually fitted to Shannon diversity, richness, and CLR-transformed abundance of each OTU as the response variables. Fixed effects were breastfeeding frequency, 24 h breastfeeding duration, and 24 h milk removal from the sampled breast. Full model outputs are reported in Supplementary Table 1. Associations between these variables and beta diversity were assessed by PERMANOVA using the adonis2 function of the vegan R package^23^ with a backward selection model with 999 permutations. P-values of < 0.05 were considered significant for all statistical analyses. R (version 4.3.1) was used for all statistical analysis and data visualisation^24^.

## Electronic supplementary material

Below is the link to the electronic supplementary material.

Supplementary Material 1

## Data Availability

Sequence data have been deposited in NCBI’s SRA (BioProject accession: PRJNA1229851).
