# Supplementary material for: The human milk microbiome is minimally associated with breastfeeding practices
Source: Sci Rep. 2025 Jun 2;15:19308. doi: 10.1038/s41598-025-03907-7 (PMC12130265; doi:10.1038/s41598-025-03907-7)
Supplement: Supplementary file 1 — Supplementary Material 1 [file 41598_2025_3907_MOESM1_ESM.pdf]

The human milk microbiome is minimally associated with breastfeeding practices

Ruomei Xu[1,2,3], Mark P. Nicol[2,4], Ali S. Cheema[5], Jacki L. McEachran[1,2,3], Ashleigh H. Warden[1,2,3], Sharon L. Perrella[1,2,3], Zoya Gridneva[1,2,3], Donna T. Geddes[1,2,3], Lisa F. Stinson[1,2,3,*]

1 School of Molecular Sciences, The University of Western Australia, Crawley, WA 6009, Australia
2 UWA Centre for Human Lactation Research and Translation, Crawley, WA 6009, Australia
3 ABREAST Network, Perth, WA 6000, Australia
4 Marshall Centre, School of Biomedical Sciences, The University of Western Australia, Crawley, WA 6009, Australia
5 The Kids Research Institute Australia, Nedlands, WA 6009, Australia

*Corresponding author:

Lisa Stinson:

lisa.stinson@uwa.edu.au

+61 8 6488 7006

The University of Western Australia,
35 Stirling Hwy, Crawley, Western Australia, 6009, Australia.

Supplementary Table 1: Outputs of the linear models for the associations between breastfeeding characteristics (from the sampled breast) and the human milk microbiota. Significant P-values are highlighted in bold.

Response	Variable	Estimate	Standard Error	P-value
**Shannon diversity**	Breastfeeding frequency	0.032	0.053	0.544
	Breastfeeding duration	0.001	0.002	0.482
	Removed milk volume	-0.001	0.001	0.378
**OTU Richness**	Breastfeeding frequency	-1.973	2.600	0.451
	Breastfeeding duration	0.153	0.098	0.126
	Removed milk volume	-0.014	0.028	0.625
**Otu000004**	Breastfeeding frequency	-0.102	0.265	0.703
Streptococcus mitis	Breastfeeding duration	0.005	0.010	0.611
	Removed milk volume	0.002	0.003	0.597
**Otu000001**	Breastfeeding frequency	0.182	0.318	0.569
Streptococcus salivarius	Breastfeeding duration	0.026	0.012	**0.035**
	Removed milk volume	0.000	0.003	0.897
**Otu000003**	Breastfeeding frequency	0.223	0.383	0.563
Cutibacterium acnes	Breastfeeding duration	-0.025	0.015	0.090
	Removed milk volume	0.006	0.004	0.181
**Otu000002**	Breastfeeding frequency	-0.306	0.317	0.339
Staphylococcus epidermidis	Breastfeeding duration	0.015	0.012	0.206
	Removed milk volume	0.002	0.003	0.627
**Otu000005**	Breastfeeding frequency	0.243	0.272	0.377
Ralstonia pickettii	Breastfeeding duration	-0.006	0.010	0.545
	Removed milk volume	0.004	0.003	0.141
**Otu000006**	Breastfeeding frequency	0.011	0.490	0.983
Streptococcus lactarius	Breastfeeding duration	0.006	0.019	0.756
	Removed milk volume	0.010	0.005	0.070
**Otu000007**	Breastfeeding frequency	-0.312	0.395	0.434
Burkholderia contaminans	Breastfeeding duration	-0.003	0.015	0.844

	Removed milk volume	0.007	0.004	0.132
**Otu000008** *Rothia mucilaginosa*	Breastfeeding frequency	0.354	0.407	0.387
	Breastfeeding duration	-0.004	0.015	0.779
	Removed milk volume	0.000	0.004	0.957
**Otu000009** *Streptococcus parasanguinis*	Breastfeeding frequency	0.389	0.455	0.397
	Breastfeeding duration	0.002	0.017	0.910
	Removed milk volume	-0.006	0.005	0.198
**Otu000011** *Acinetobacter johnsonii*	Breastfeeding frequency	-0.325	0.333	0.333
	Breastfeeding duration	0.003	0.013	0.821
	Removed milk volume	-0.001	0.004	0.693
**Otu000014** *Veillonella* sp.	Breastfeeding frequency	0.462	0.452	0.311
	Breastfeeding duration	0.014	0.017	0.422
	Removed milk volume	-0.004	0.005	0.383
**Otu000016** *Bifidobacterium longum* subsp. *infantis*	Breastfeeding frequency	0.008	0.274	0.977
	Breastfeeding duration	0.009	0.010	0.395
	Removed milk volume	0.000	0.003	0.953
**Otu000012** *Enterobacter* sp.	Breastfeeding frequency	0.415	0.350	0.240
	Breastfeeding duration	-0.015	0.013	0.250
	Removed milk volume	-0.005	0.004	0.236
**Otu000010** *Gemella haemolysans*	Breastfeeding frequency	-0.057	0.452	0.900
	Breastfeeding duration	0.011	0.017	0.525
	Removed milk volume	0.000	0.005	0.982
**Otu000051** *Paracoccus marcusii*	Breastfeeding frequency	-0.195	0.175	0.270
	Breastfeeding duration	-0.004	0.007	0.585
	Removed milk volume	-0.002	0.002	0.215
**Otu000013** *Pseudomonas* sp. 1	Breastfeeding frequency	0.006	0.368	0.986
	Breastfeeding duration	-0.004	0.014	0.790
	Removed milk volume	-0.003	0.004	0.503
**Otu000021**	Breastfeeding frequency	-0.451	0.306	0.147

Lactobacillus sp.	Breastfeeding duration	0.022	0.012	0.066
	Removed milk volume	-0.001	0.003	0.695
**Otu000023** *Bradyrhizobium* sp.	Breastfeeding frequency	0.306	0.398	0.445
	Breastfeeding duration	0.008	0.015	0.604
	Removed milk volume	-0.001	0.004	0.905
**Otu000025** *Pelomonas* sp.	Breastfeeding frequency	0.153	0.394	0.700
	Breastfeeding duration	0.004	0.015	0.793
	Removed milk volume	0.001	0.004	0.875
**Otu000036** *Pseudomonas* sp. 2	Breastfeeding frequency	0.111	0.209	0.596
	Breastfeeding duration	0.001	0.008	0.889
	Removed milk volume	-0.003	0.002	0.137

Supplementary Table 2: Species-level taxonomic assignments from BLAST. In cases where >1 species was an equally good match, genus-level taxonomy was used here, and all matching species are described in this table.

OTU	Taxonomy	% ID	% Coverage	Accession
Otu000001	*Streptococcus salivarius*	98.25%	100%	CP145862.1
Otu000002	*Staphylococcus epidermidis*	99.12%	100%	CP064619.1
Otu000003	*Cutibacterium acnes*	98.55%	100%	CP012647.1
Otu000004	*Streptococcus mitis*	98.65%	100%	CP133471.1
Otu000005	*Ralstonia pickettii*	98.56%	100%	CP066771.1
Otu000006	*Streptococcus lactarius*	98.64%	100%	CP072329.1
Otu000007	*Burkholderia contaminans*	98.97%	100%	CP120947.1
Otu000008	*Rothia mucilaginosa*	98.83%	100%	CP097094.1
Otu000009	*Streptococcus parasanguinis*	98.57%	100%	CP134147.1
Otu000010	*Gemella haemolysans*	99.53%	100%	CP083637.1
Otu000011	*Acinetobacter johnsonii*	99.11%	100%	CP068206.1
Otu000012	*Enterobacter asburiae*	99.11%	100%	CP162150.1
	Enterobacter roggenkampii	99.05%	100%	CP128618.1
Otu000013	*Pseudomonas putida*	98.70%	100%	CP047150.1
	Pseudomonas juntendi	98.77%	100%	CP079903.1
Otu000014	*Veillonella dispar*	99.33%	100%	LR134375.1
	Veillonella nakazawae	99.33%	100%	AP022321.1
Otu000016	*Bifidobacterium longum subsp. infantis*	98.50%	100%	AP010889.1
Otu000021	*Lactobacillus paragasseri*	98.93%	100%	CP141798.1
	Lactobacillus gasseri	98.93%	100%	CP177271.1
Otu000023	*Bradyrhizobium septentrionale*	98.60%	100%	CP147708.1
	Bradyrhizobium quebecense	98.60%	100%	CP088282.1
Otu000025	*Pelomonas* sp.	98.62%	100%	KF441635.1
Otu000036	*Pseudomonas fragi*	98.91%	100%	CP129917.1
	Pseudomonas bubulae	98.98%	100%	CP146077.1
Otu000051	*Paracoccus marcusii*	98.92%	100%	CP157011.1

Supplementary Table 3: Genera detected from negative extraction (EC) and negative amplification (NTC) controls. Data are sequence counts.

**Genus**	**EC1**	**EC2**	**EC3**	**EC4**	**EC5**	**EC6**	**EC7**	**NTC 1**	**NTC 2**	**NTC 3**	**NTC 4**	**NTC 5**	**NTC 6**	**NTC 7**
Acinetobacter	0	1	0	0	0	0	1	0	0	0	0	0	0	1
Unclassified *Actinobacteria*	0	2	0	0	0	0	1	1	1	0	0	1	2	1
Actinomyces	0	1	0	0	1	0	0	0	0	0	1	0	0	0
Alloprevotella	0	0	1	0	0	0	0	0	0	0	0	0	0	0
Unclassified *Alphaproteobacteria*	0	1	0	0	0	0	0	0	0	0	0	0	0	0
Anaerococcus	0	0	1	1	0	0	0	0	0	0	0	0	0	0
Anthococcus	0	0	0	0	0	0	0	1	0	0	0	0	0	0
Azomonas	0	0	0	0	0	0	0	0	0	0	1	0	0	0
Unclassified *Bacillaceae*	0	1	0	0	0	0	0	0	0	0	0	0	0	0
Unclassified *Bacillales*	2	3	0	1	0	1	0	0	4	0	0	0	0	1
Unclassified *Bacilli*	2	3	0	0	1	0	1	0	0	0	3	1	0	2
Bacillus	1	0	0	0	0	0	0	1	0	0	0	0	0	0
Unclassified *Bacteria*	2	12	0	1	0	0	3	3	4	2	1	2	2	2
Bergeyella	0	0	0	0	0	0	2	0	0	0	0	0	0	2
Unclassified *Betaproteobacteriales*	0	2	0	0	1	0	0	1	0	0	0	0	0	0
Unclassified *Bifidobacteriaceae*	0	0	0	0	0	0	0	0	0	0	0	0	0	1
Bifidobacterium	0	0	0	0	0	0	0	0	1	0	0	0	1	0
Bradyrhizobium	0	0	2	0	6	1	0	0	0	0	1	0	0	0
Burkholderia-Caballeronia-Paraburkholderia	8	0	0	3	3	2	0	1	1	1	1	2	2	0
Unclassified *Burkholderiaceae*	3	0	0	0	3	1	0	1	1	1	0	0	1	0
Unclassified *Carnobacteriaceae*	0	0	0	0	0	0	0	0	0	1	0	0	0	1
Unclassified *Caulobacteraceae*	0	1	0	0	0	0	0	0	0	0	0	0	0	0
Unclassified *Cellulomonadaceae*	1	0	0	0	0	0	0	0	0	0	0	0	0	0
Unclassified *Corynebacteriaceae*	0	0	2	0	0	0	0	0	0	0	0	1	0	0
Corynebacterium	0	1	0	1	0	0	0	0	0	0	0	1	1	0
Cutibacterium	3	1	1	0	1	0	0	3	4	0	3	0	1	0
Dolosigranulum	1	0	0	0	0	0	0	0	1	0	0	0	0	0
Dyadobacter	0	1	0	0	0	0	0	0	0	0	0	0	0	0
Endobacter	0	1	0	0	0	0	0	0	0	0	0	0	0	0

Enhydrobacter	0	0	0	0	0	0	0	1	0	1	0	0	0	0
Enterobacter	0	0	1	0	0	0	0	0	0	0	0	0	0	0
Unclassified *Enterobacteriaceae*	0	0	0	0	0	0	0	0	0	0	2	0	0	0
F0332	0	0	0	0	0	0	0	0	1	0	0	0	0	0
Fibrisoma	0	3	0	0	0	0	0	0	0	0	0	0	0	0
Flavisolibacter	0	3	0	0	0	0	0	0	0	0	0	0	0	0
Unclassified *Flavobacteriaceae*	0	2	0	0	0	0	0	0	0	0	0	0	0	0
Unclassified *Gammaproteobacteria*	0	1	0	0	0	0	0	0	0	0	0	0	0	0
Gemella	1	0	0	0	1	1	1	0	0	0	1	0	3	1
Granulicatella	1	1	0	0	0	1	2	0	2	1	1	1	0	2
Haemophilus	0	0	1	0	0	0	0	0	0	0	1	0	0	0
Halomonas	0	0	0	0	0	1	0	0	0	0	0	0	0	0
JGI_0000069-P22	0	0	0	0	0	0	0	0	0	0	0	0	0	1
Unclassified *Lactobacillales*	3	4	1	0	0	0	2	2	0	1	0	0	0	0
Lactobacillus	0	0	1	1	1	0	1	0	0	0	0	0	0	0
Leptotrichia	0	0	0	0	0	0	1	0	0	0	0	0	0	0
Unclassified *Leptotrichiaceae*	0	0	0	0	0	0	0	0	0	0	0	0	0	1
Mesorhizobium	0	0	0	1	0	0	0	0	0	0	0	0	0	0
Unclassified *Microbacteriaceae*	0	2	0	0	0	0	0	0	0	0	0	0	0	0
Unclassified *Micrococcaceae*	0	0	0	0	0	0	3	0	0	1	1	0	0	0
Unclassified *Micrococcales*	0	0	0	0	0	0	1	0	0	1	0	0	0	1
Unclassified *Myxococcales*	0	1	0	0	0	0	0	0	0	0	0	0	0	0
Neisseriaceae	0	0	0	0	0	0	0	0	0	0	0	0	0	1
Unclassified *Neisseriaceae*	0	0	0	0	0	1	1	0	0	0	0	0	0	0
Niveispirillum	0	1	0	0	0	0	0	0	0	0	0	0	0	0
Niveitalea	0	1	0	0	0	0	0	0	0	0	0	0	0	0
Oligotropha	0	0	1	0	0	0	0	0	0	0	0	1	0	0
Unclassified *Oxyphotobacteria*	0	1	0	0	0	0	0	0	0	0	0	0	0	0
Paracoccus	3	0	0	0	0	0	0	0	0	0	0	0	0	0
Unclassified *Pasteurellaceae*	0	1	0	0	0	0	0	0	1	0	0	0	0	1
Pelomonas	0	0	1	0	1	0	0	0	0	1	0	0	0	0
Peptoniphilus	0	0	0	0	0	1	0	0	0	0	0	0	0	0
Prevotella_7	0	0	0	0	0	0	0	0	0	0	0	0	0	1
Unclassified *Propionibacteriaceae*	0	3	0	1	1	0	0	0	1	0	0	0	1	0

Unclassified *Proteobacteria*	0	2	0	0	0	0	0	0	0	0	0	0	0	0
Unclassified *Pseudomonadaceae*	2	1	0	1	0	0	0	0	0	0	0	0	0	0
Pseudomonas	0	4	0	2	0	0	0	0	0	0	0	0	0	0
Psychrobacter	0	0	0	0	0	0	1	0	0	0	0	0	0	0
Ralstonia	15	4	2	6	5	3	0	1	1	1	0	1	0	0
Unclassified *Rhizobiaceae*	0	1	0	1	0	0	0	0	0	0	0	0	0	0
Unclassified *Rhodobacteraceae*	0	0	0	0	0	0	1	0	0	0	0	0	0	0
Rothia	1	1	2	1	1	0	2	1	1	2	0	1	1	9
Rubinisphaera	0	3	0	0	0	0	0	0	0	0	0	0	0	0
Saccharimonadaceae	0	1	0	0	0	0	0	0	0	0	0	0	0	0
Unclassified *Sphingomonadaceae*	0	1	0	0	1	0	0	0	0	0	0	0	0	0
Sphingomonas	0	0	0	0	2	0	0	0	0	0	0	0	0	0
Sporocytophaga	0	0	0	0	0	0	0	0	1	0	0	0	0	0
Unclassified *Staphylococcaceae*	1	0	0	0	1	0	0	0	0	0	0	0	0	0
Staphylococcus	4	2	3	2	1	2	0	4	3	1	3	2	1	1
Unclassified *Streptococcaceae*	1	2	0	1	0	0	1	1	0	0	0	0	0	2
Streptococcus	15	14	10	10	11	3	5	13	10	4	7	3	9	10
uncultured	0	2	0	0	0	1	0	0	0	0	0	0	0	0
Veillonella	2	2	1	1	0	0	1	1	1	0	0	0	0	0
Unclassified *Veillonellaceae*	0	1	1	1	0	0	1	0	0	0	1	0	0	0
Unclassified *Weeksellaceae*	2	0	0	0	0	0	0	0	0	0	0	0	0	0
Unclassified *Xanthobacteraceae*	0	0	0	0	1	0	0	0	0	1	0	0	0	0

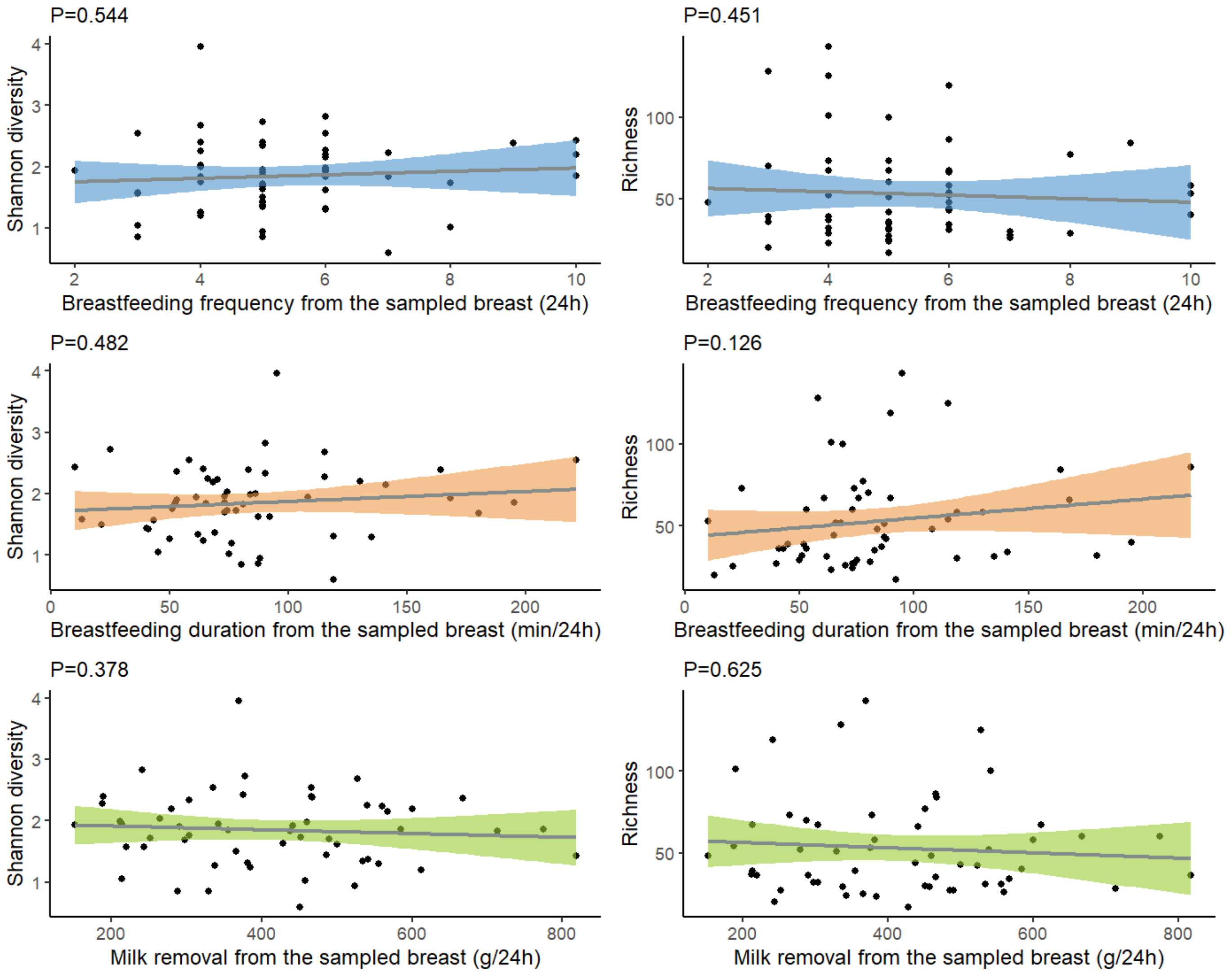

Supplementary Figure 1: Breastfeeding characteristics (data from the sampled breast only) were not associated with Shannon diversity or richness of the human milk microbiome. Lines represent linear models fitted to the data with shaded areas representing 95% confidence interval
